# First Chinese ultraviolet–visible hyperspectral satellite instrument implicating global air quality during the COVID-19 pandemic in early 2020

**DOI:** 10.1038/s41377-022-00722-x

**Published:** 2022-02-02

**Authors:** Cheng Liu, Qihou Hu, Chengxin Zhang, Congzi Xia, Hao Yin, Wenjing Su, Xiaohan Wang, Yizhou Xu, Zhiguo Zhang

**Affiliations:** 1 Department of Precision Machinery and Precision Instrumentation, University of Science and Technology of China, 230026 Hefei, China; 2 Key Laboratory of Environmental Optics and Technology, Anhui Institute of Optics and Fine Mechanics, Hefei Institutes of Physical Science, Chinese Academy of Sciences, 230031 Hefei, China; 3 Center for Excellence in Regional Atmospheric Environment, Institute of Urban Environment, Chinese Academy of Sciences, 361021 Xiamen, China; 4 Key Laboratory of Precision Scientific Instrumentation of Anhui Higher Education Institutes, University of Science and Technology of China, 230026 Hefei, China; 5 School of Earth and Space Sciences, University of Science and Technology of China, 230026 Hefei, China; 6 Department of Environmental Science and Engineering, University of Science and Technology of China, 230026 Hefei, China

**Keywords:** Optical spectroscopy, Atmospheric optics

## Abstract

In response to the COVID-19 pandemic, governments worldwide imposed lockdown measures in early 2020, resulting in notable reductions in air pollutant emissions. The changes in air quality during the pandemic have been investigated in numerous studies via satellite observations. Nevertheless, no relevant research has been gathered using Chinese satellite instruments, because the poor spectral quality makes it extremely difficult to retrieve data from the spectra of the Environmental Trace Gases Monitoring Instrument (EMI), the first Chinese satellite-based ultraviolet–visible spectrometer monitoring air pollutants. However, through a series of remote sensing algorithm optimizations from spectral calibration to retrieval, we successfully retrieved global gaseous pollutants, such as nitrogen dioxide (NO_2_), sulfur dioxide (SO_2_), and formaldehyde (HCHO), from EMI during the pandemic. The abrupt drop in NO_2_ successfully captured the time for each city when effective measures were implemented to prevent the spread of the pandemic, for example, in January 2020 in Chinese cities, February in Seoul, and March in Tokyo and various cities across Europe and America. Furthermore, significant decreases in HCHO in Wuhan, Shanghai, Guangzhou, and Seoul indicated that the majority of volatile organic compounds (VOCs) emissions were anthropogenic. Contrastingly, the lack of evident reduction in Beijing and New Delhi suggested dominant natural sources of VOCs. By comparing the relative variation of NO_2_ to gross domestic product (GDP), we found that the COVID-19 pandemic had more influence on the secondary industry in China, while on the primary and tertiary industries in Korea and the countries across Europe and America.

## Abstract (web summary)

The changes of global gaseous pollutants during the COVID-19 pandemic in early 2020 were retrieved from Chinese ultraviolet–visible hyperspectral satellite instrument through a series of remote sensing algorithm optimizations.

## Graphical abstract

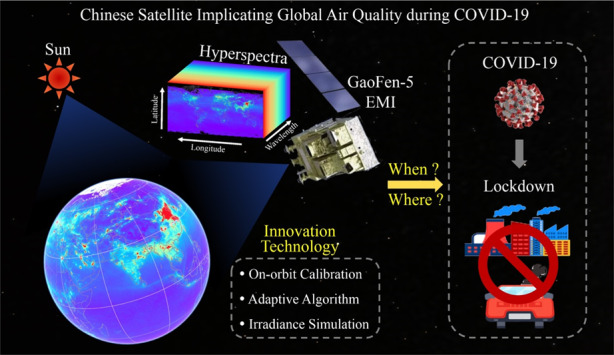

## Introduction

Since early 2020, the novel coronavirus (COVID-19), a severe infectious disease, began to spread worldwide. In response to the COVID-19 pandemic, governments worldwide implemented special measures (lockdowns) to prevent crowd gathering, which have resulted in a global reduction in air pollutant emissions^1,2^. Numerous studies have been conducted to investigate the variations in air quality owing to the pandemic. For instance, some studies have focused on the significant reduction in atmospheric nitrogen dioxide (NO_2_) from space-borne observations by high-resolution instruments, such as the Tropospheric Monitoring Instrument (TROPOMI) and Ozone Monitoring Instrument (OMI)^3,4^, based on in-situ monitoring^5,6^, and through atmospheric chemical transport modeling^7,8^. Satellite-based remote sensing has a distinctive advantage when compared to surface observations, especially in terms of spatial coverage and data consistency, which enable us to easily compare air quality discrepancies in different countries. For instance, Bauwens et al.^3^ and Sun et al.^9^ explored global changes in the levels of NO_2_ and formaldehyde (HCHO) through satellite observations.

The Environmental Trace Gases Monitoring Instrument (EMI) onboard the GaoFen-5 satellite is the first Chinese satellite-based ultraviolet–visible hyperspectral spectrometer (spectral resolution of <0.6 nm). Along with TROPOMI and OMI, it is also a space-borne high-resolution instrument for air pollutant observation. Because the original spectral quality of EMI is considerably inferior to that of TROPOMI, it has thus far not been used for air quality research during the COVID-19 pandemic. However, by conducting a series of optimizations from spectral calibration to inversion settings in our previous studies^10,11^, a comparable final inversion data quality with TROPOMI was achieved. In this study, we used the tropospheric vertical column densities (TVCDs) of multiple gaseous pollutants observed by EMI to investigate the variations, potentially caused by lockdown measures, in air pollutants such as NO_2_, sulfur dioxide (SO_2_), and HCHO across different countries and cities. In addition, the relationships between the pandemic, economy, and air quality are discussed.

## Results

### Retrieval of NO_2_, SO_2,_ and HCHO from EMI

Launched in May 2018, EMI onboard the Gaofen-5 satellite is a push-broom spectrometer with ultraviolet and visible spectral bands from 240 to 710 nm and a nadir spatial resolution of 12 × 13 km^2^. The basic retrieval methods for EMI are similar to those for OMI and TROPOMI. The NO_2_ TVCDs are retrieved in three steps. First, the total NO_2_ slant column density (SCD) is retrieved using the differential optical absorption spectroscopy (DOAS) technique. As suggested by the QA4ECV NO_2_ project^12^, the absorption cross-section of ozone (O_3_), NO_2_, oxygen dimer (O_4_), water vapor, and liquid water, as well as ring effect, were considered. Subsequently, the tropospheric NO_2_ SCD is obtained after separating stratospheric NO_2_ through the STRatospheric Estimation Algorithm from Mainz^13^. Finally, air mass factors (AMFs) are used to convert tropospheric SCDs to tropospheric VCDs (TVCDs).

1 $${{{\mathrm{VCD}}}} = {{{\mathrm{SCD}}}}/{{{\mathrm{AMF}}}}$$

For HCHO retrieval, the first step is the retrieval of the differential slant column density (DSCD) by fitting radiances through the basic optical differential spectroscopy (BOAS) method. DSCD represents the difference between the true SCD value and SCD in the reference spectra (the daily earth radiance in the remote Pacific Ocean). Cross-sections of O_3_ at two temperatures, NO_2_, O_4,_ and bromine monoxide (BrO) were considered in the retrieval of HCHO DSCDs^14^. Then, the DSCD is converted to SCD through reference sector correction, because earth radiance in the remote Pacific Ocean contains a small amount of HCHO absorption^14–16^. Similar to the conversion of NO_2_, the final step is the conversion of HCHO SCD to HCHO VCD using AMF. As HCHO is concentrated in the lower troposphere, the retrieved HCHO VCD is approximately equal to the HCHO TVCD. Further, because EMI shares the same overpass time as TROPOMI, in the AMF calculation for NO_2_ and HCHO retrieval, parameters including cloud information and surface albedo are from TROPOMI. Simulated profiles of NO_2_ and HCHO by the GEOS-Chem model are used as a priori profiles in the AMF calculation.

SO_2_ TVCDs are retrieved using the optimal estimation (OE) algorithm by minimizing the cost function. It simultaneously considers the difference between the simulated and measured reflectance and the difference between the retrieved and the a priori state vectors. The fitting is constrained by the measurement and a priori uncertainty covariance matrix. The vector linearized discrete ordinate radiative transfer model (VLIDORT) is then employed to simulate the radiance containing only Rayleigh scattering and O_3_ absorption. The Lambert-Beer Laws introduce other trace gases effects with the a priori profiles from the GEOS-Chem simulations. Cross-sections of O_3_ at four temperatures, BrO and HCHO were considered for SO_2_ retrieval^17^.

**Fig. 1:**
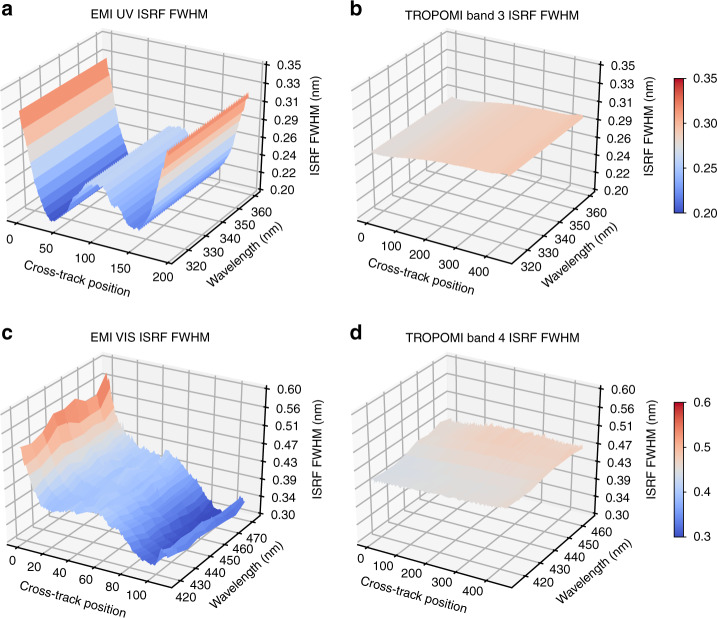
Comparison of ISRF FWHM for EMI and TROPOMI in ultraviolet and visible spectra. **a** The on-orbit ISRF FWHM of EMI in ultraviolet spectra. **b** Similar to **a**, but for the preflight TROPOMI band 3 spectra. **c** Similar to **a**, but for the on-orbit EMI visible spectra. **d** Similar to **a**, but for the preflight TROPOMI band 4 spectra.

**Fig. 2:**
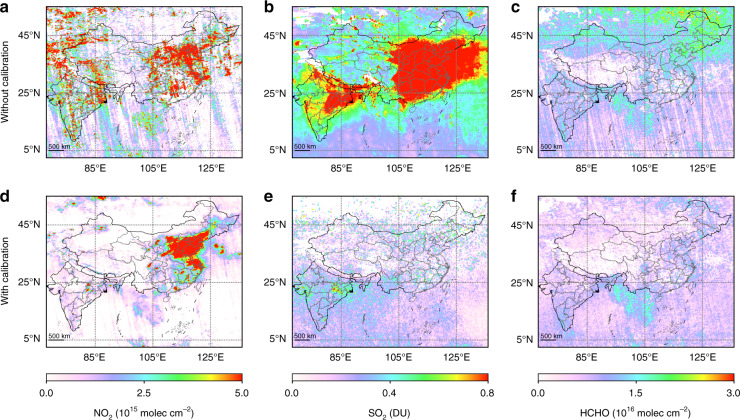
Comparison of EMI trace gas retrievals in March 2020 with and without spectral calibration. **a–c** The retrieval of NO_2_, SO_2_, and HCHO without spectral calibration, respectively; **d–f** The retrieval of NO_2_, SO_2_, and HCHO with spectral calibration, respectively.

However, because of the poor original spectral quality of EMI (Fig. 1), the retrieval results are noisy and show unrealistic distributions without pre-calibrations (Fig. 2a–c). Therefore, we developed several new methods to improve the traditional retrieval.

1. The full width at half maximum (FWHM) of the EMI instrumental spectral response functions (ISRFs) changes much more drastically than that of the TROPOMI ISRFs in spatial and temporal dimensions (Fig. 1), because the EMI instrument lacks preflight ISRFs and the instrument performance is degraded by the complex space environment. Simultaneously, the EMI wavelength shifts are much larger (Fig. S1 in the Supplementary Information). To address this problem, we performed on-orbit wavelength calibration to calculate daily ISRFs and wavelength shifts to diminish the fitting residuals.
2. The signal-to-noise ratio (SNR) of EMI is very low, at only one-third of that of TROPOMI. Therefore, there was a need to minimize the interference of other trace gases in the retrieval fitting windows and reduce the fitting residual under such a low SNR of EMI. Here we set up an adaptive iterative retrieval algorithm that selects the retrieval setting best with minimum uncertainty among fitting results using different retrieval settings. The retrieval settings not only contain the fitting windows but also consider low-order polynomials and different trace gas parameters, etc. For instance, instead of directly using wavelength fitting ranges suggested in the retrievals from OMI and TROPOMI in previous studies, the wavelength fitting ranges were chosen by the adaptive iterative retrieval algorithm as 420–470 nm, 326.5–356 nm, and 310.5–320 nm for NO_2_, HCHO, and SO_2_ retrievals from EMI, respectively.
3. Owing to the insufficient mechanical structure of optical path switching, EMI only provides the solar spectrum once every 6 months. In addition, EMI provides abnormal irradiance measurements owing to a diffuser calibration issue. To obtain the requisite daily solar spectrum for the following retrieval algorithm, we used simulated irradiance instead of using measured irradiance. This eliminated the cross-track stripes in the retrieval and reduced the average fitting residuals.

Further, retrieval results were improved by obtaining algorithm updates (Fig. 2d–f). In particular, the spectra for SO_2_ retrieval contain more noise because the strongest SO_2_ absorption band (300–330 nm) is close to the edge of the measured spectra. Therefore, traditional retrieval settings greatly overestimate the SO_2_ TVCDs in China and India^11^. Improved SO_2_ data were obtained through the above three measures, as well as extra measurement error correction and pixel merging (Fig. 2e). The calibrated data from EMI were validated by surface observations in India and TROPOMI data retrieved using our improved algorithm^11^. The detailed retrieval algorithms for NO_2_, SO_2,_ and HCHO from EMI are described in our previous studies^10,11,18^.

According to the uncertainty propagation, the systematic error of the NO_2_ TVCD is due to systematic uncertainty in SCD retrieval (<3%), AMF calculation (15–40%), and stratospheric separation (<10%). This caused a total systematic error of NO_2_ TVCDs of ~18% in the summer and 42% in the winter^10,12^. The systematic error of the HCHO VCD is due to systematic uncertainty in SCD retrieval (~17%), AMF calculation (15–51%), and reference sector correction (10–40%)^14,19^. Combined, these produce a total uncertainty of ~25% in clean regions and 67% in polluted regions. Uncertainty from the AMF calculation contributes the most to the systematic error of NO_2_ and HCHO TVCDs. The parameters used in the EMI AMF calculation are similar to the TROPOMI AMF calculation, and the systematic errors in these two retrievals are of the same order of magnitude. The SO_2_ concentration in the remote Pacific Ocean (10° S-10° N, 135°−90° W) is assumed to be zero. Therefore, the retrieved SO_2_ TVCD in the remote Pacific region is identified as the systematic error of SO_2_ TVCD retrieval, which is ~0.29 DU^11^.

**Fig. 3:**
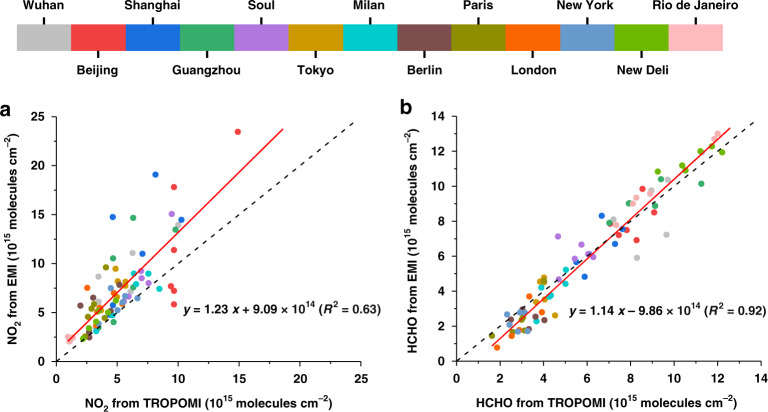
Comparisons of NO_2_ and HCHO TVCDs from EMI and TROPOMI. Correlation analysis of the monthly (**a**) NO_2_ TVCDs and **b** HCHO TVCDs from EMI and TROPOMI in the first quarter of 2019 and 2020 in Wuhan, Beijing, Shanghai, Guangzhou, Seoul, Tokyo, Milan, Berlin, Paris, London, New York, New Delhi, and Rio de Janeiro. NO_2_ and HCHO TVCDs in a city were calculated as the average of TVCDs in satellite pixels within a radius of 50 km around the city center

Nevertheless, systematic retrieval errors will not affect the comparison of TVCDs in the years 2019 and 2020. However, the random error can be used to evaluate whether the satellite observation can capture the monthly variability of the pollutants^20^. The random retrieval error of air pollutants in the investigated city was calculated as $\overline {{{{\mathrm{error}}}}} /\sqrt {n_{{{{\mathrm{pix}}}}}}$, where $\overline {{{{\mathrm{error}}}}}$ is the average random retrieval error of all the satellite pixels over the city and n_pix_ is the number of pixels over the city^20^. Therefore, spatial sampling is the main factor influencing random error. The monthly TVCDs of NO_2_ and HCHO retrieved from EMI showed good consistency with those from the operational products of TROPOMI (Fig. 3), although the absolute values from the two instruments had some deviation and the random retrieval errors of HCHO from EMI were larger than those from TROPOMI. We did not compare the SO_2_ results from the two satellite instruments because of the systemic deviation of the operational SO_2_ products from TROPOMI^21^.

### Global air quality variations during COVID-19 pandemic from EMI observation

**Fig. 4:**
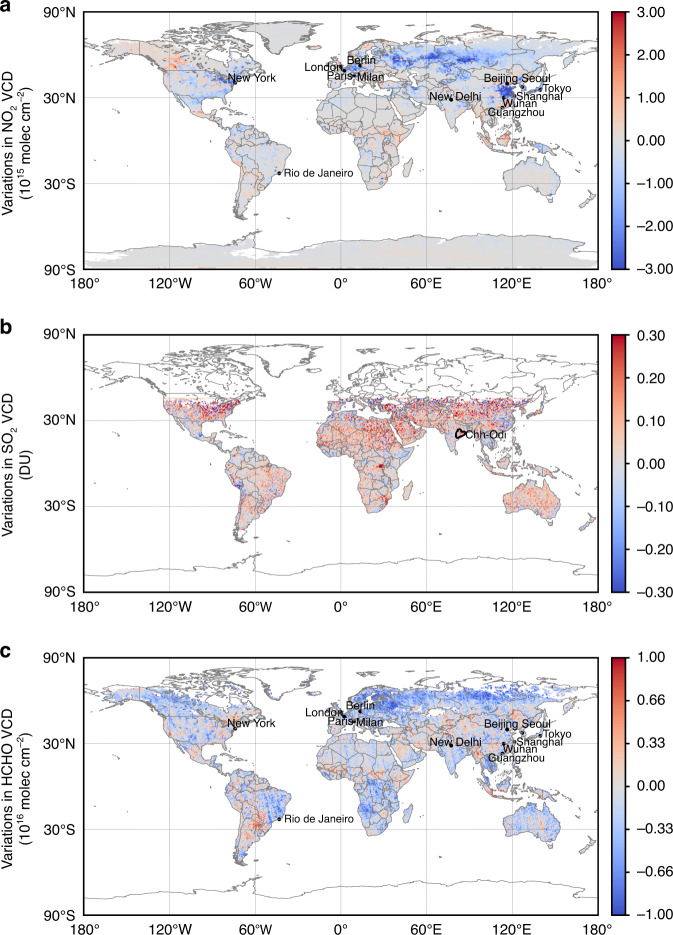
Spatial distributions of the variations in air pollutants. The variations in the average TVCDs based on EMI observation of **a** NO_2_, **b** SO_2_, and **c** HCHO from March 2019 to March 2020.

Nitrogen oxides (NO_x_ = NO + NO_2_) are short-lived trace gas species produced by combustion emissions, such as coal combustion for power generation and industrial production, and from vehicle exhausts^22,23^. NO_2_ TVCD retrieved from satellite observations has been widely used to indicate anthropogenic emissions^24,25^. In March 2020, when the COVID-19 pandemic had spread all over the world, the global average NO_2_ TVCD from EMI observations was 1.3 × 10^14^ molecules cm^−2^ (20%) lower than that in March 2019. The reductions in NO_2_ TVCD were more evident in regions with high concentrations, such as eastern China, western Europe, and eastern North America (Fig. 4a). In most investigated cities, the monthly NO_2_ TVCDs showed good consistency with surface concentrations (Fig. S2). However, in Tokyo, Berlin, and Paris, the surface concentrations showed relatively narrower variations. Similar trends in the column and surface concentrations further confirmed the decrease in NO_x_ emissions. In particular, when governments implemented active measures to prevent the spread of the pandemic, NO_2_ TVCD accordingly decreased abruptly^4^. Therefore, the changes in NO_2_ TVCD can reflect the response to the pandemic in different cities; this will be discussed in detail in the following section.

In addition to NO_2_, SO_2_ is also an important anthropogenic pollutant from power generation, oil refineries, smelters, and domestic heating^26,27^. In contrast to the significant reduction of NO_2_, SO_2_ TVCDs in March 2020 showed a slightly increasing global trend compared to that in March 2019 (Fig. 4b). With the application of desulfurization techniques, SO_2_ emissions have greatly decreased worldwide in recent years, and apart from in India, atmospheric SO_2_ worldwide dropped dramatically^28,29^. Obvious SO_2_ emission hotspots were only observable in India through satellite observation^11^. SO_2_ TVCDs from EMI in most of the other regions were noisy. Therefore, we specifically tracked the changes in SO_2_ in the Chhattisgarh-Odisha region in India, where various large coal-fired power plants are gathered and SO_2_ concentrations are among the highest in the world. When the pandemic struck India in March 2020, the observed SO_2_ TVCD was slightly higher than that in March 2019 (Fig. S3), and the random retrieval errors did not exceed the changes in the SO_2_ TVCDs. Furthermore, because the concentration of air pollutants can also be affected by meteorology, we adjusted the SO_2_ TVCDs in 2019 to those under the meteorological conditions of 2020 (deweathered SO_2_) according to GEOS-Chem modeling (seeing method). The results showed that the SO_2_ TVCD in March 2020 was much higher than the deweathered TVCD in March 2019, indicating that the power industry was unaffected by the pandemic until then. Other studies had similar findings. For instance, Zhang et al.^30^ found no obvious reductions in SO_2_ TVCDs in India during the first quarter of 2020 when compared to the first quarter of years 2015–2019. Instead, great reductions in SO_2_ levels were noted in April and May, likely due to the implementation of strict lockdown measures from late March. In addition, Shi et al.^31^ found no substantial change in SO_2_ in Chinese cities of Wuhan and Beijing during the lockdown period, further indicating that the main sources of SO_2_, such as residential heating and energy production, were not as easily affected by the pandemic as those of NO_2_^31^.

HCHO in ambient air is formed by the atmospheric chemical reaction of non-methane hydrocarbons (NMHCs)^32,33^, as well as through direct emissions from anthropogenic activities and biomass burning^34–36^. HCHO TVCDs from satellite-based remote sensing has been widely used to trace VOC emissions^37–39^. The global average of HCHO TVCD in March 2020 was 1.5 × 10^15^ molecules cm^−2^ (21%) lower than that in March 2019. However, distinct variations were observed in the different regions (Fig. 4c). Similar variations in global NO_2_ and HCHO between March 2019 and March 2020 were also observed by TROPOMI (Fig. S4).

### The change of NO_2_ from EMI observation indicates the lockdown measures in different cities

**Fig. 5:**
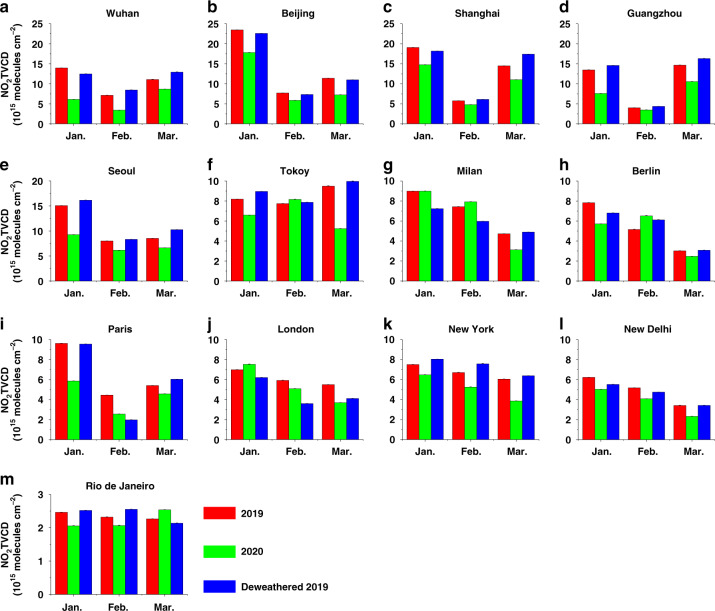
Comparisons of NO_2_ TVCDs in 2019 and 2020. Monthly TVCDs of NO_2_ in 2019 (red) and 2020 (green), and deweathered TVCDs of NO_2_ in 2019 (blue) in **a** Wuhan, **b** Beijing, **c** Shanghai, **d** Guangzhou, **e** Seoul, **f** Tokyo, **g** Milan, **h** Berlin, **i** Paris, **j** London, **k** New York, **l** New Delhi, and **m** Rio de Janeiro based on EMI observation. The black bars are the random retrieval errors.

The monthly increase in infections of COVID-19 in China proliferated in January and reached a peak in February 2020 (Fig. S5). The COVID-19 pandemic first broke out in Wuhan, China, and the city has been under an emergent lockdown measure since January 23, 2020. Although the measure only covered nine days in January, the monthly NO_2_ TVCD in Wuhan from EMI observation, which was calculated as the average of TVCDs in all the satellite pixels within a radius of 50 km around the city center, decreased by 56% relative to that in January 2019 (Fig. 5a). Even after deducting the influence of meteorology, the NO_2_ TVCD in January 2020 decreased by 51%. In addition, the random retrieval errors were less than 1% of the monthly TVCDs, which were much lower than the change in NO_2_ TVCDs. Therefore, the sharp variation in NO_2_ TVCD in January 2020 can be attributed to a significant reduction in emissions. The annual one-week-long Chinese New Year (CNY) holiday occurred in January of 2020 but in February of 2019. Therefore, the decline in NO_2_ from January 2019 to January 2020 included the influence of the holiday effect. By directly comparing NO_2_ TVCDs during the CNY week in both years, we found a decrease of 1.2 × 10^16^ molecules cm^−2^ (77%) from 2019 to 2020. This could not be fully explained by meteorological conditions, further proving the role of lockdown on ambient NO_2_. Following Wuhan, other cities in China, such as Beijing, Shanghai, and Guangzhou, also established restrictive lockdown measures before January 26. The deweathered reduction percent of NO_2_ in January for Guangzhou was comparable with that for Wuhan; however, the reductions for Beijing and Shanghai were much lower (Fig. 5b–d). This follows the conclusion put forth by Ding et al.^40^, who suggested no significant reductions of NO_x_ emissions in Beijing in January 2020. A similar reduction trend for NO_2_ TVCDs was also observed by TROPOMI (Fig. S6).

Among the sources of NO_x_, lockdown measures first affected transportation, followed by industry. However, because basic living needs must be met, energy production should not be drastically reduced. Indeed, residential emissions may have increased because more people remain at home^31^. According to the emission inventory from the Community Emissions Data System (CEDS)^41^, transportation contributed about 33% of the NO_x_ emissions in Wuhan. Alone, this does not cover the reduction of NO_2_ in January, suggesting that most industries were also shut down, causing the reduction of NO_x_ in Wuhan to reach an ultimate level without affecting basic living needs. Contrastingly, the decline in NO_2_ did not exceed the transportation contribution in Beijing, Shanghai, and Guangzhou.

In February 2020, lockdown measures were continuously implemented in China. During this period, NO_2_ concentrations across the four cities in China further decreased from those in January 2020; they were also significantly lower than those in February 2019. The largest reduction in the NO_2_ TVCDs still occurred in Wuhan. Because of the effective measures, the increase in COVID-19 infections dropped sharply in March 2020, and except for Wuhan, the lockdown was lifted in China. Since then, productivity and living activities in Wuhan have also gradually recovered. Therefore, NO_2_ concentrations in the four cities significantly increased from February to March 2020. However, the concentrations in the four cities remained approximately 30% lower than those in March 2019.

In February 2020, the COVID-19 pandemic also spread across Korea and Japan, and the spread in Korea was noted to be faster than that in Japan. The Korean government placed restrictions to prevent the gathering of people and strengthen nucleic acid detection. The restriction measures caused a decrease in NO_2_ TVCD by 26% in Seoul when compared with the deweathered TVCD in February 2019 (Fig. 5e). In contrast, no lockdown measures were taken by Japan in February, suggesting a reason for the unaffected NO_2_ TVCD in Tokyo (Fig. 5f). The pandemic in Korea and Japan was further aggravated in March 2020. The NO_2_ TVCD in Seoul further decreased to 35% lower than the deweathered value in 2019, which is consistent with the decrease in surface NO_2_ concentration in this city^42^. In addition, owing to more stringent social distancing measures, the NO_2_ TVCD in Tokyo also sharply decreased.

In Europe, the COVID-19 pandemic first broke out in Italy in March 2020 and also reached its peak during that month. On March 10, the Italian government implemented strict measures to prevent gatherings. Corresponding to this period in March 2020, the recorded NO_2_ TVCD in Milan was 34% lower than that in March 2019, with meteorological corrections (Fig. 5g). EMI data was not available from April 2020, because of the failure in the solar battery of the Gaofen-5 satellite. Thus, after March 2020, we analyzed the changes in NO_2_ based on TROPOMI observations. The NO_2_ TVCD in Milan in April showed a declining trend because of continued lockdown (Fig. S6). In addition to column concentration, ground-level NO_2_ in this city also decreased by ~30% during the lockdown^43^. In the UK, Germany, and France, lockdown measures were issued from the middle and late March. The average NO_2_ TVCDs in Berlin and Paris in March 2020 significantly decreased compared with the deweathered TVCDs in March 2019, but the reduction percentage was much lower than that for Milan (Fig. 5h–i). However, because of the later implementation of lockdown measures in the UK, the deweathered reduction percent of NO_2_ TVCD in London in March was less than 10% (Fig. 5j). Surface observation in the UK also showed a more gradual decline of NO_2_ early in the outbreak of COVID-19 when compared to the sudden decline in the other European countries^6^. Apart from Italy, the other three European countries faced increasing detected infections of COVID-19 in April. The NO_2_ TVCDs with meteorological corrections in London and Paris during this month decreased by 38% and 48%, respectively. This indicated that restriction measures in both cities were strictly implemented in April, although the pandemic had broken and the lockdown measures had been established in March. During this month, the reduction in Paris even exceeded the contribution of transportation.

In the US, the pandemic broke out in March and then rapidly spread until June; lockdown measures were taken from late March. Correspondingly, NO_2_ TVCD decreased by 40% compared with the deweathered TVCD in March 2019 (Fig. 5k), which was slightly higher than the contribution of transportation. However, these measures were not mandatory, and gradually loosened until they were entirely relieved. As such, the NO_2_ TVCDs showed no evident reduction after April (Fig. S6).

In India and Brazil, the COVID-19 pandemic struck in March 2020 and broke out in April; lockdown measures were implemented from late March. In March 2020, NO_2_ TVCD decreased by 32% and 19% in New Delhi and Rio de Janeiro, respectively, when compared with the deweathered TVCDs in March 2019 (Fig. 4l–m). However, to recover the economy, the lockdown measures were gradually released from late May despite the more severe pandemic. Therefore, the NO_2_ TVCDs in both cities in June recovered to comparable levels in the corresponding month of 2019 (Fig. S6).

### HCHO change indicates the relative importance of anthropogenic and natural sources of VOCs

**Fig. 6:**
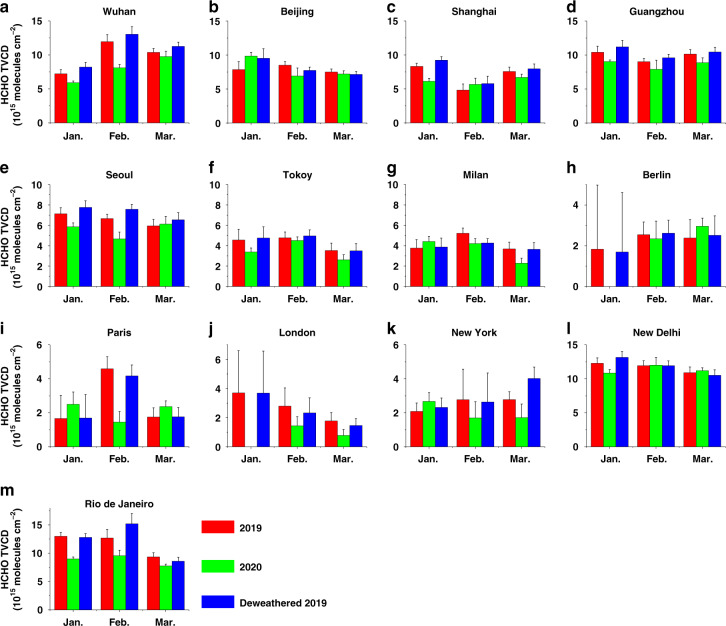
Comparisons of HCHO TVCDs in 2019 and 2020. Similar to Fig. 5, but for HCHO.

The COVID-19 pandemic has been suggested to abate anthropogenic emissions of VOCs but not biogenic emissions. Therefore, changes in HCHO can indicate whether the VOCs in a city mainly originate from anthropogenic or biogenic sources. Monthly HCHO TVCDs from EMI observations in Wuhan from January to March 2020 were significantly lower than those in the corresponding months of 2019, with the maximum reduction seen in February (Figs. 6 and S7). Though Wuhan was very often covered by clouds in February 2019, which led to less spatial sampling and larger random error of HCHO TVCD than that in February 2020, the errors did not exceed the variations in HCHO TVCDs. A similar phenomenon was observed in Guangzhou and Shanghai. This suggests that anthropogenic emissions are a major source of VOCs and were substantially affected in these three cities. Lower HCHO yields from NMHC with NO_x_ emission plunges may also reduce HCHO TVCDs^9^. However, the HCHO TVCDs in Beijing in January and March 2020 were even higher than those during the corresponding periods of 2019, and the decrease in HCHO TVCDs in February was close to the random retrieval errors. This may be explainable as enterprises with high VOC emissions (e.g., chemical plants) were moved to Hebei Province in 2014 to improve air quality in Beijing. As such, it can be assumed that most VOCs in Beijing originates from biogenic emissions, which were not disturbed by the pandemic.

HCHO in the cities of the other investigated countries also showed different trends. In Milan, Berlin, and London, HCHO only presented a significant decrease in the first one or two months after the pandemic broke out but remained at comparable levels following this, with the random retrieval errors being considered (Figs. 6 and S7). One reason for this phenomenon is that the contribution of VOCs from biogenic sources is likely to have increased with an increase in temperature from winter to spring. HCHO in Tokyo and Rio de Janeiro decreased synchronously with NO_2_ at the beginning of lockdown, but the reduction percentage was lower than that of NO_2_ in the following months. In Seoul, the reduction in HCHO levels was comparable to that in NO_2_ levels; however, in New Delhi, no evident reduction in HCHO levels was observed. It should be considered, however, that various complex sources can cause distinct variations in HCHO levels. For instance, Sun et al.^9^ reported that the HCHO trends in Australia, northeastern Myanmar of Southeast Asia, Central Africa, and Central America were dominated by fire activities during the pandemic.

## Discussion

The attenuation of anthropogenic activities during the pandemic caused a decline in both gross domestic product (GDP) and air pollutant emissions. Here, we use the ratio of quarterly averaged NO_2_ TVCD to the quarterly GDP in a country (NO_2_/GDP) to indirectly reflect NO_2_ emissions per unit GDP. As NO_x_ emissions are mainly from the secondary industry, such as manufacturing and mining, the convergence between the variations of GDP and NO_2_ TVCDs can reveal whether the decline in GDP was mainly from the secondary industry, or the primary and tertiary industries, such as agriculture and service trade. To compare the changes in NO_2_/GDP in different countries, we calculated the relative variation of NO_2_ to GDP (△(NO_2_/GDP)) as follows:

2 $$\begin{array}{ll}{\Delta}\left( {{{{\mathrm{NO}}}}_2/{{{\mathrm{GDP}}}}} \right) = \left(\right. {{{\mathrm{NO}}}}_2\;{{{\mathrm{TVCD}}}}_{2020}/{{{\mathrm{GDP}}}}_{2020}\\ \qquad\qquad\qquad\qquad- {{{\mathrm{NO}}}}_2\;{{{\mathrm{TVCD}}}}_{{{{\mathrm{deweathered}}}}\;2019}/{{{\mathrm{GDP}}}}_{2019} \left)\right.\\ \qquad\qquad\qquad\qquad /\left( {{{{\mathrm{NO}}}}_2\;{{{\mathrm{TVCD}}}}_{{{{\mathrm{deweathered}}}}\;2019}/{{{\mathrm{GDP}}}}_{2019}} \right)\end{array}$$

Here, NO_2_ TVCD_2020_ is the quarterly averaged NO_2_ TVCD in 2020, and NO_2_ TVCD_deweathered 2019_ is the deweathered quarterly NO_2_ TVCD in 2019. GDP_2019_ and GDP_2020_ are quarterly data for 2019 and 2020, respectively.

**Fig. 7:**
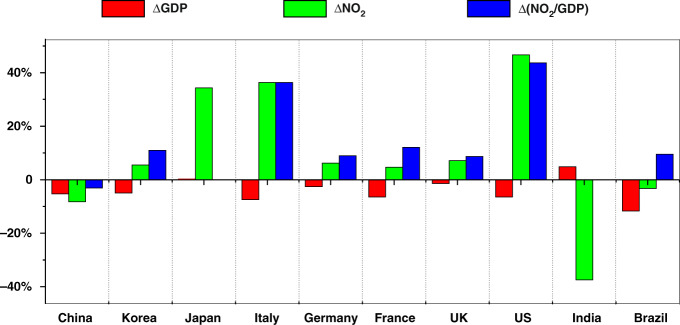
The change percentage of GDP (△GDP), NO_2_ (△NO_2_), and the ratio of NO_2_ to GDP (△(NO_2_/GDP)) from the first quarter of 2019 to the first quarter of 2020 for the ten investigated countries. NO_2_ TVCD was adopted from EMI observation, and those for 2019 have been deweathered. As the GDPs for Japan and India were not obviously decreased, △(NO_2_/GDP) was not calculated for the two countries.

In the first quarter of 2020, the GDP in eight of the ten investigated countries, except for India and Japan, significantly decreased. Quarterly NO_2_ TVCDs decreased in the three developing countries, China, India, and Brazil, but increased in the other seven countries despite a substantial reduction in March. In China, △(NO_2_/GDP) was below 0, indicating that the reduction percent of NO_2_ TVCD was more than GDP, therefore suggesting that the secondary industry was more affected by the pandemic (Fig. 7). The higher NO_2_/GDP values in the remaining seven countries suggested that the decline in GDP was caused primarily due to impacts on the primary and tertiary industries. As such, this study proves that Chinese satellite instruments can play an important role in the global monitoring of atmospheric environmental events.

## Materials and methods

### TROPOMI data

TVCDs of NO_2_ and HCHO from TROPOMI were also used in this study. TROPOMI onboard the Sentinel-5 Precursor (S-5P) was launched in October 2017. It measures ultraviolet and visible backscattered radiances from 266 to 775 nm, with a spectral resolution of 0.5 nm. The nadir spatial resolution of TROPOMI was 7 × 3.5 km^2^ which was then, improved to 3.5 × 5.5 km^2^ after August 2019^44^. The data from TROPOMI was directly accessed via the Sentinel-5P Pre-Operations Data Hub website.

### Correction of meteorological influence using GEOS-Chem Modeling

The GEOS-Chem model, a global 3-D chemical transport model (CTM), was used to deduce the influence of meteorology on air pollutant concentrations by simulating the change in air pollutants from 2019 to 2020. This model is driven by the Goddard Earth Observing System (GEOS) assimilated meteorological fields from the NASA Global Modeling and Assimilation Office^45^. The model includes a detailed mechanism of the universal tropospheric-stratospheric chemistry extension (UCX) mechanism^46^. Further, global anthropogenic and biofuel emissions from the CEDS inventory^41^ were used and processed through the Harvard-NASA emission component (HEMCO)^47^. NO_2_, SO_2,_ and HCHO in 2019 and 2020 were simulated using the same inventory and their respective meteorological fields in different years. The observed TVCDs of these pollutants in 2019 were adjusted to those under the meteorological conditions in 2020 (TVCD_deweathered 2019_) as follows:

3 $$\begin{array}{ll}{{{\mathrm{TVCD}}}}_{{{{\mathrm{deweathered}}}}\;2019} = {{{\mathrm{TVCD}}}}_{{{{\mathrm{obs}}}}\;2019} \\ \qquad \qquad \qquad \qquad \qquad \times \,{{{\mathrm{TVCD}}}}_{{{{\mathrm{modeling}}}}\;2020}/{{{\mathrm{TVCD}}}}_{{{{\mathrm{modeling}}}}\;2019}\end{array}$$

Here, TVCD_obs 2019_ is the observed TVCDs from satellite instruments in 2019, and TVCD_modeling 2019_ and TVCD_modeling 2020_ are the simulated TVCDs from GEOS-Chem for the years of 2019 and 2020, respectively.

## Supplementary information

Supplementary Information

## Data Availability

The EMI level 1 and NO_2_, SO_2_, and HCHO datasets are available from Cheng Liu (chliu81@ustc.edu.cn) and Qihou Hu (qhhu@aiofm.ac.cn) upon reasonable request. The TROPOMI NO_2_ and HCHO datasets are available from https://s5phub.copernicus.eu/dhus/#/home.
