# Supplementary material for: First Chinese ultraviolet–visible hyperspectral satellite instrument implicating global air quality during the COVID-19 pandemic in early 2020
Source: Light Sci Appl. 2022 Feb 2;11:28. doi: 10.1038/s41377-022-00722-x (PMC8809219; doi:10.1038/s41377-022-00722-x)
Supplement: Supplementary file 1 — Supplementary Information [file 41377_2022_722_MOESM1_ESM.pdf]

Supplementary Information for the manuscript:

First Chinese ultraviolet–visible hyperspectral satellite instrument implicating global air quality during the COVID-19 pandemic in early 2020

Cheng Liu[1,2,3,4], Qihou Hu[2,*], Chengxin Zhang[1], Congzi Xia[5], Hao Yin[2], Wenjing Su[6], Xiaohan Wang[1], Yizhou Xu[1], Zhiguo Zhang[1]

[1] Department of Precision Machinery and Precision Instrumentation, University of Science and Technology of China, Hefei 230026, China;

[2] Key Laboratory of Environmental Optics and Technology, Anhui Institute of Optics and Fine Mechanics, Hefei Institutes of Physical Science, Chinese Academy of Sciences, Hefei, 230031, China

[3] Center for Excellence in Regional Atmospheric Environment, Institute of Urban Environment, Chinese Academy of Sciences, Xiamen 361021, China

[4] Key Laboratory of Precision Scientific Instrumentation of Anhui Higher Education Institutes, University of Science and Technology of China, Hefei 230026, China

[5] School of Earth and Space Sciences, University of Science and Technology of China, Hefei 230026, China

[6] Department of Environmental Science and Engineering, University of Science and Technology of China, Hefei 230026, China

[*]Corresponding author. E-mail: qhhu@aiofm.ac.cn.

To complement the information provided in the main manuscript, the following sections provide supporting information (Figures S1-S7).

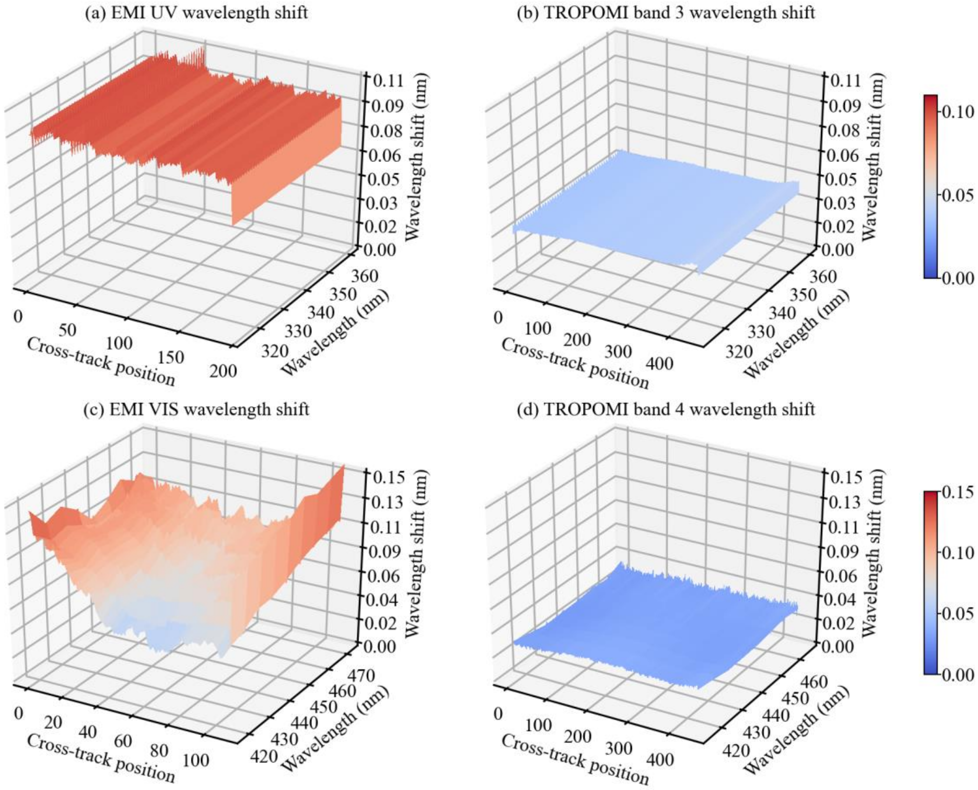

Figure S1. Same as Figure 1, but for the comparison of wavelength shift.

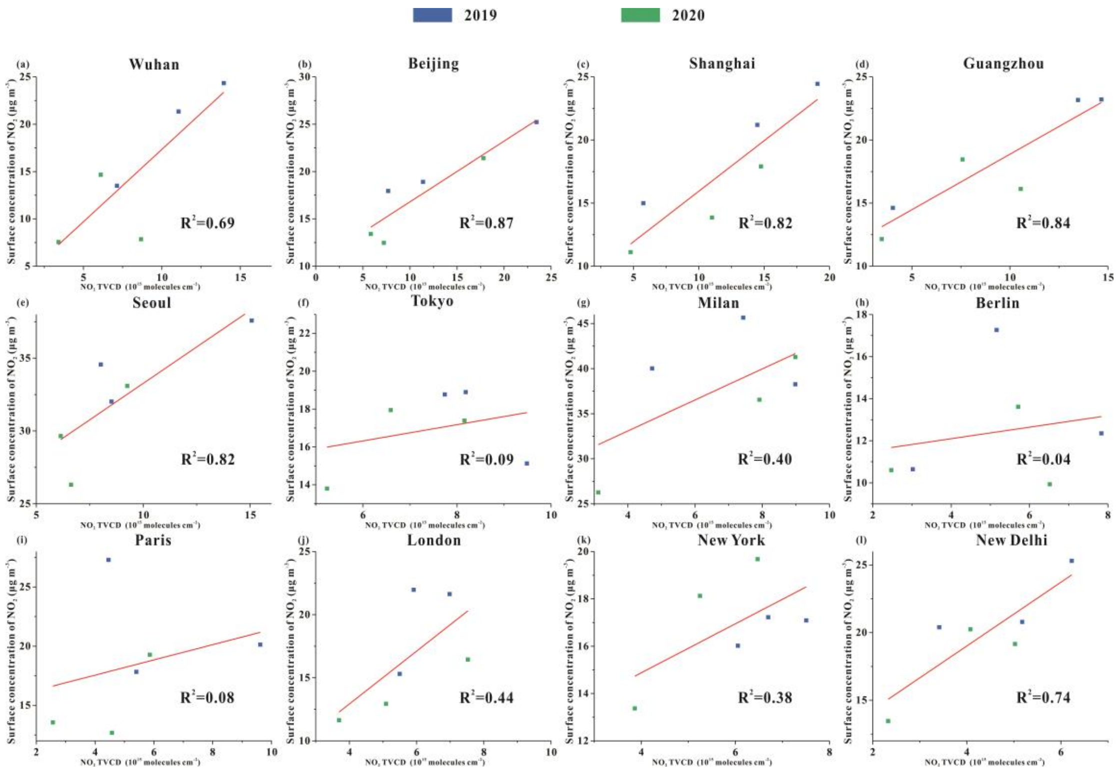

Figure S2. Comparison of the monthly NO_2 TVCDs from EMI and the surface concentrations of NO_2 in (a) Wuhan, (b) Beijing, (c) Shanghai, (d) Guangzhou, (e) Seoul, (f) Tokyo, (g) Milan, (h) Berlin, (i) Paris, (j) London, (k) New York and (l) New Delhi. The blue squares indicate the data in 2019 and the green squares indicate the data in 2020. The surface NO_2 concentrations were obtained from the *World Air Quality Index* (https://aqicn.org/here/). No available insitu NO_2 data was found for Rio de Janeiro.

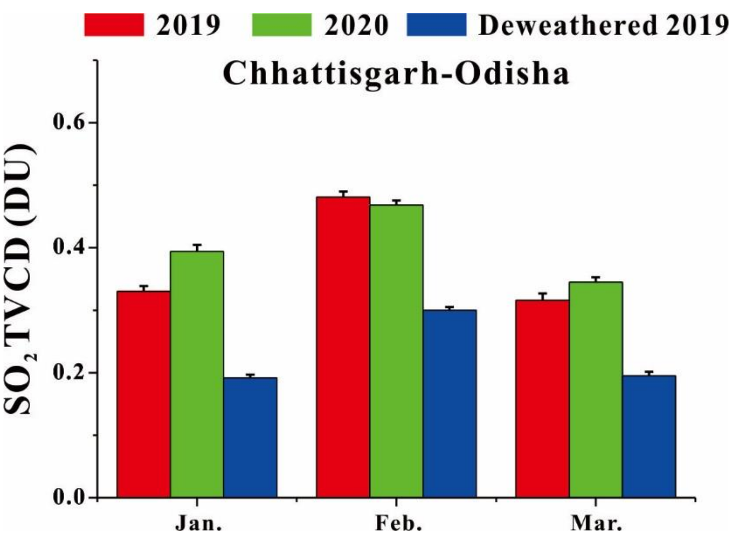

Figure S3. Similar to Figure 5, but for SO_2 in the Chhattisgarh-Odisha region in India.

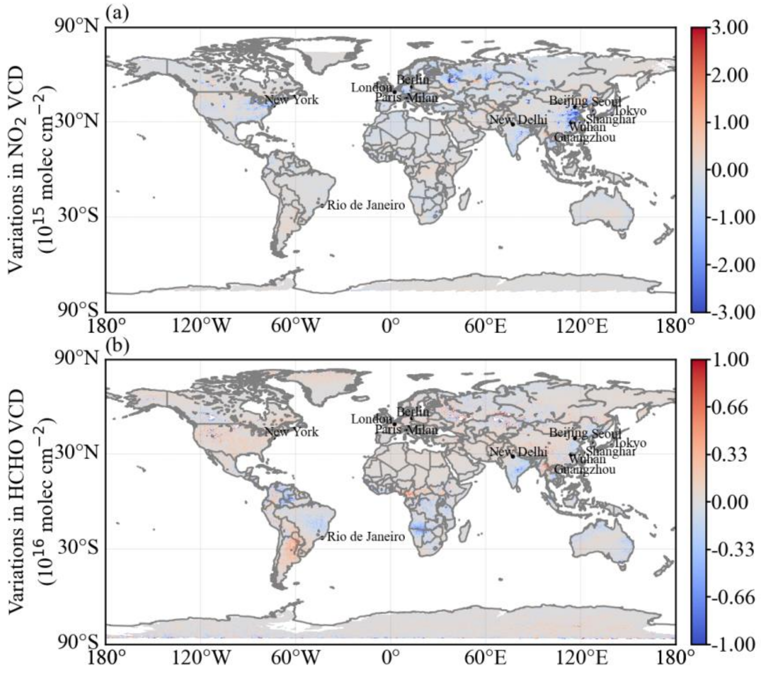

Figure S4. Similar to Figure 4, but the NO_2 and HCHO TVCDs data were taken from TROPOMI observations.

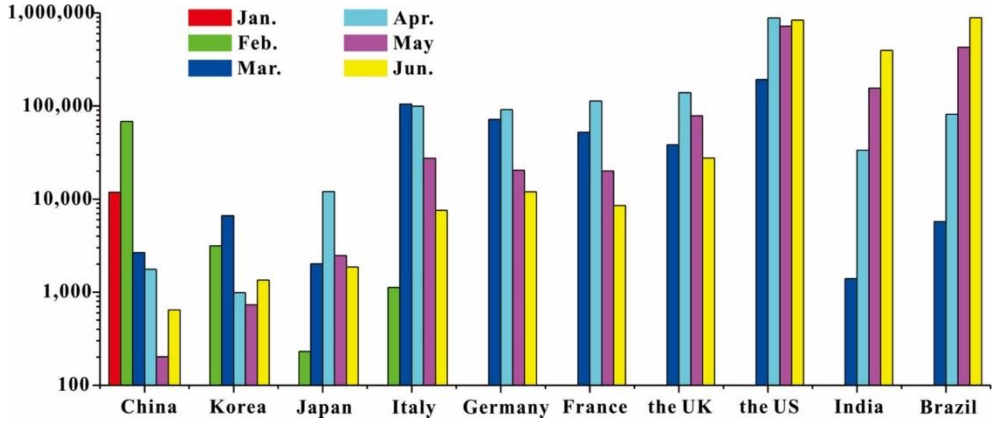

Figure S5. Monthly increase in infections of COVID-19 for the ten investigated countries.

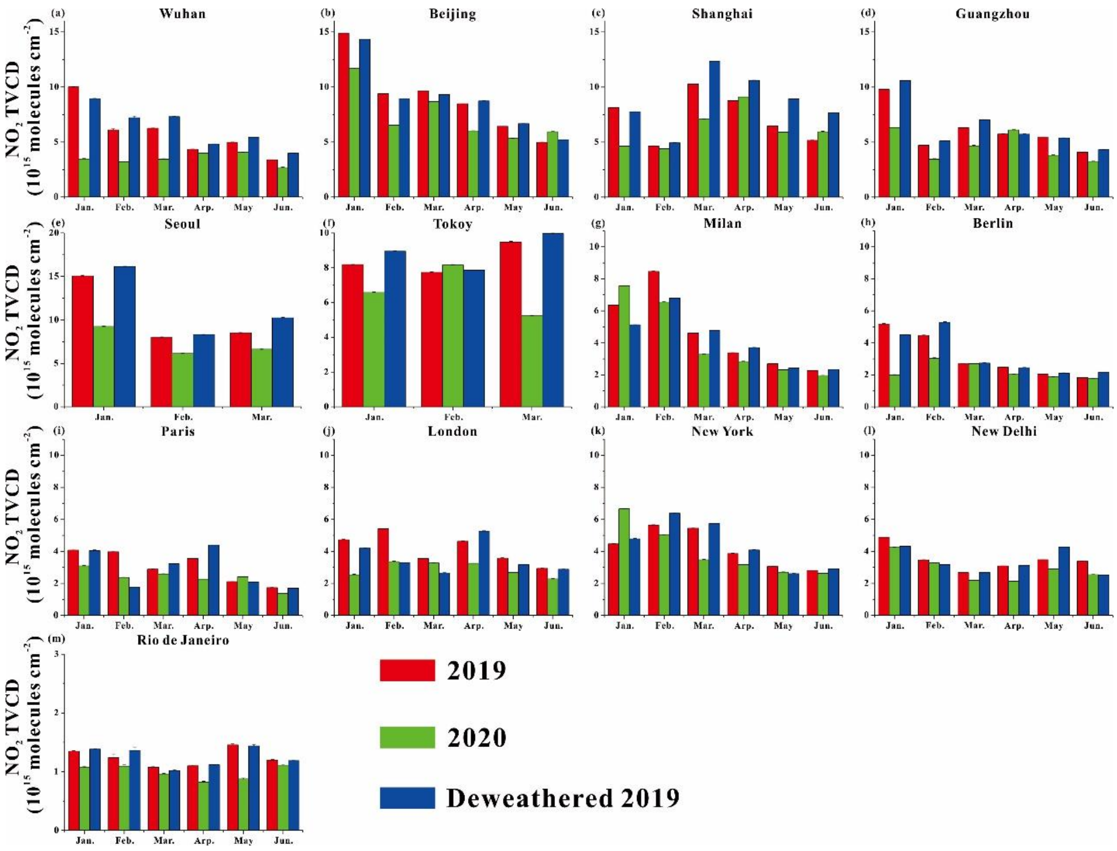

Figure S6. Similar to Figure 5, but the NO_2 TVCD data was from TROPOMI observations.

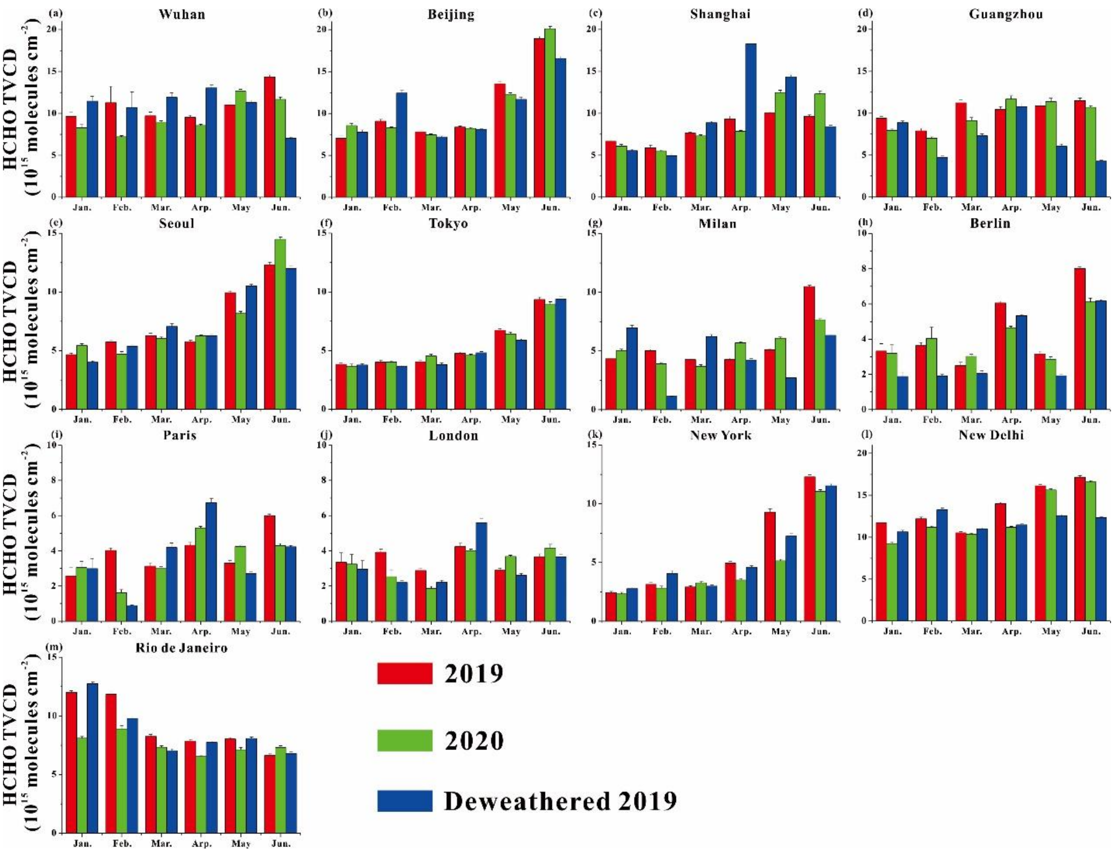

Figure S7. Similar to Figure 6, but the HCHO TVCD data was from TROPOMI observations.
